# Integrating Tobacco Control and Obesity Prevention Initiatives at Retail Outlets

**DOI:** 10.5888/pcd13.150426

**Published:** 2016-03-10

**Authors:** Kurt M. Ribisl, Heather D’Angelo, Kelly R. Evenson, Sheila Fleischhacker, Allison E. Myers, Shyanika W. Rose

**Affiliations:** Author Affiliations: Kurt M. Ribisl, Heather D’Angelo, Allison E. Myers, Gillings School of Global Public Health, Department of Health Behavior, University of North Carolina, Chapel Hill, North Carolina; Kelly R. Evenson, Gillings School of Global Public Health, Department of Epidemiology, University of North Carolina, Chapel Hill, North Carolina; Sheila Fleischhacker, National Institutes of Health, Division of Nutrition Research Coordination, Bethesda, Maryland; Shyanika W. Rose, Schroeder Institute for Tobacco Research and Policy Studies at Truth Initiative, Washington, DC. Dr Ribisl is also affiliated with the Lineberger Comprehensive Cancer Center, University of North Carolina, Chapel Hill, North Carolina.

## Abstract

Tobacco products are sold in approximately 375,000 US retail outlets, including convenience stores and pharmacies, which often sell energy-dense, low-nutrient foods and beverages. The Food and Drug Administration’s (FDA’s) increased authority over tobacco product sales and marketing, combined with declining smoking rates, provides an opportunity to transition tobacco retailers toward healthier retail environments. Unfortunately, research into improving consumer retail environments is often conducted in isolation by researchers working in tobacco control, nutrition, and physical activity. Interdisciplinary efforts are needed to transform tobacco retailers from stores that are dependent on a declining product category, to the sale and promotion of healthful foods and creating environments conducive to active living. The objective of this article is to describe the potential for interdisciplinary efforts to transition retailers away from selling and promoting tobacco products and toward creating retail environments that promote healthful eating and active living.

## Introduction

The strong decline in current cigarette smoking by adults in the United States (US) over the past 50 years (1) has occurred primarily because of policy changes, including tax increases, clean indoor air laws, bans on broadcast tobacco advertising, and stronger protections against sales to minors. The 2009 Family Smoking Prevention and Tobacco Control Act (PL 111–31, known as the “Tobacco Control Act”) has the potential to change how tobacco products are sold and marketed at retail outlets (2), many of which also sell food products. Declining smoking rates and corresponding declines in cigarette sales, combined with changes to the consumer environment at outlets that sell both food and tobacco products, create promising but time-sensitive opportunities to integrate tobacco control and obesity prevention at the point of sale. The objective of this article is to describe the potential for multisectoral efforts and policy options to encourage retailers to move away from selling, displaying, and promoting tobacco products and toward creating retail environments that promote healthful eating and active living.

The estimated 374,584 tobacco retailers in the contiguous United States (3) are a venue for implementing interventions and enacting policies to improve access to healthful food and promote physical activity opportunities. Eight major establishment types sell tobacco products (Table 1), and of these, all but tobacco stores generate greater revenue from food sales than from tobacco sales. The recommendations here are more suited to establishments that sell both high volumes of food and tobacco products, such as supermarkets, convenience stores, and gas stations. Convenience stores, particularly those located in low-income neighborhoods designated as food deserts, increasingly are sites for “healthy store” interventions to increase healthful food availability (4). Unfortunately, these convenience stores also sell and heavily market tobacco products with advertisements often targeted to young people. In addition, food deserts are often concentrated in disadvantaged neighborhoods.

**Table 1:** Tobacco and Food Sales of Establishments Selling Tobacco Products

Establishment Type	Total Sales ($1,000)	Sales of Tobacco Products ($1,000)	Sales of Food Products ($1,000)
Gasoline stations with convenience stores	336,275,435	26,765,721	35,823,951
Supermarkets and other grocery stores	466,225,948	8,126,850	349,932,807
Warehouse clubs and superstores	324,963,224	7,489,940	126,388,507
Tobacco stores	7,073,689	6,132,093	296,799
Convenience stores	20,881,468	5,112,203	9,805,630
Pharmacies and drug stores	202,042,128	1,880,373	8,795,339
Beer, wine and liquor stores	36,313,659	1,418,408	1,484,020
Other gasoline stations	114,137,626	1,361,586	2,762,870

Source: US Census Bureau, 2007 Economic Census.

## Tobacco Product Marketing and Promotion at the Point of Sale

The tobacco industry developed comprehensive strategies to promote tobacco products at the point of sale, especially in communities characterized by social and economic disadvantage (5). Cigarette manufacturers spent 92.1% of their $9.17 billion in marketing and promotional expenditures exclusively or predominantly at retail stores in 2012 (6). The average store that sells cigarettes features 29.5 tobacco product advertisements (3). As one example, industry documents detail how the Brown and Williamson “Kool Inner City Point of Purchase Program” created specific incentives, tailored advertising and promotional items, and crafted ambitious programs to provide free samples at inner-city retailers (7). Further, low-income and racial/ethnic minority neighborhoods have more tobacco advertisements at the point of sale (8) and more tobacco retailers compared with higher income, predominantly white neighborhoods (9). Thus, racial/ethnic minority and socioeconomically disadvantaged neighborhoods face the dual problem of having more tobacco retailers and being saturated with more tobacco advertising than retailers in predominantly white, higher income areas.

Point-of-sale tobacco marketing is a public health problem because it distorts adolescents’ perceptions about the availability, use, and popularity of cigarettes and promotes adolescent smoking (10). The tobacco industry focused much of its attention on the point of sale over the past decade, in part, because the retail outlet has been one of the least regulated venues for tobacco marketing and promotion. However, the Tobacco Control Act is changing how the tobacco industry communicates with consumers through point-of-sale advertising.

## Impact of the Tobacco Control Act on Tobacco Retailers

Two provisions of the Tobacco Control Act in particular would change how consumers are exposed to tobacco products and marketing at retail outlets, and retailers will need to reevaluate how the aesthetics of their store will change as a result. The typical convenience store is saturated with colorful tobacco advertisements along sidewalks and fences, in parking lots, on exterior store walls and windows, and surrounding cash registers. Under the Tobacco Control Act, the FDA is considering prohibiting outdoor cigarette and smokeless tobacco product advertising near schools and public playgrounds. If the FDA bans outdoor cigarette and smokeless tobacco advertising within 1,000 feet of schools, it could lead to the removal of nearly 1.5 million tobacco advertisements (11).

**Figure 1:**
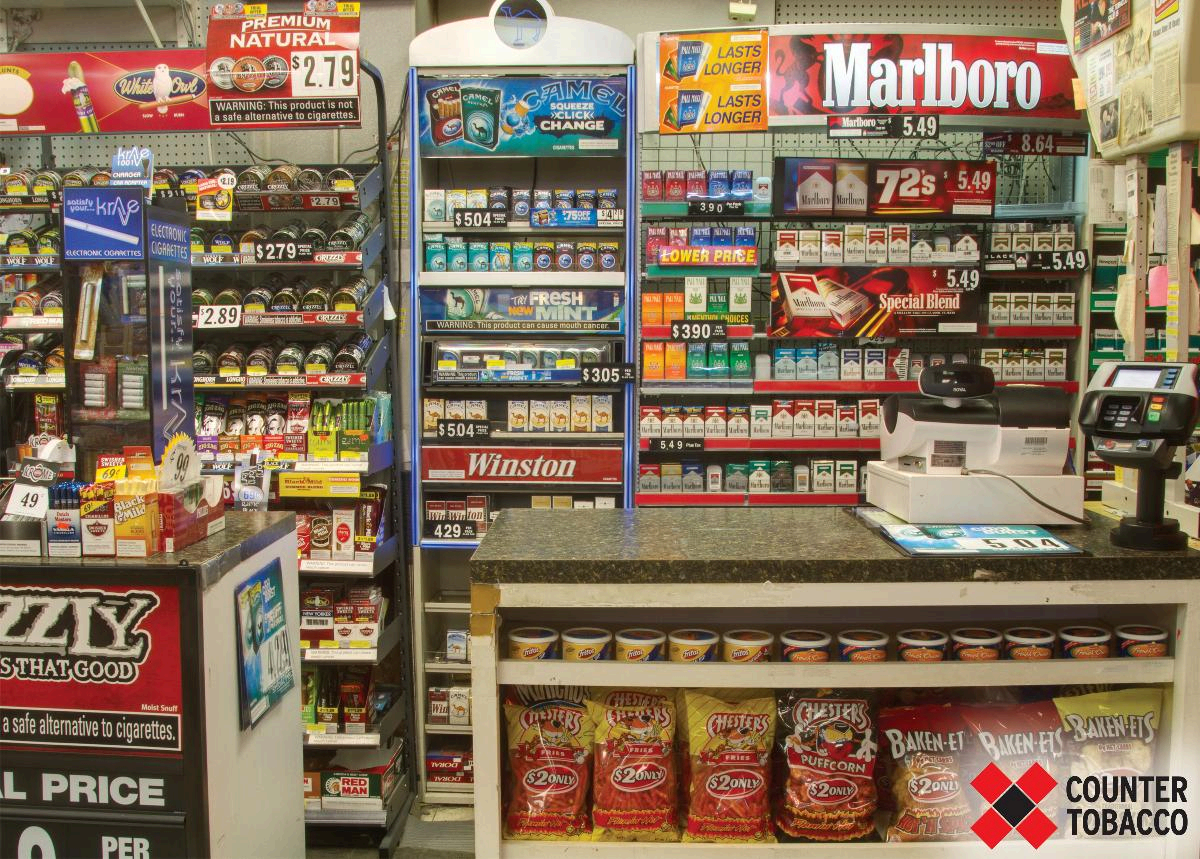
Customer view of tobacco advertising in a typical US convenience store (before).

**Figure 2:**
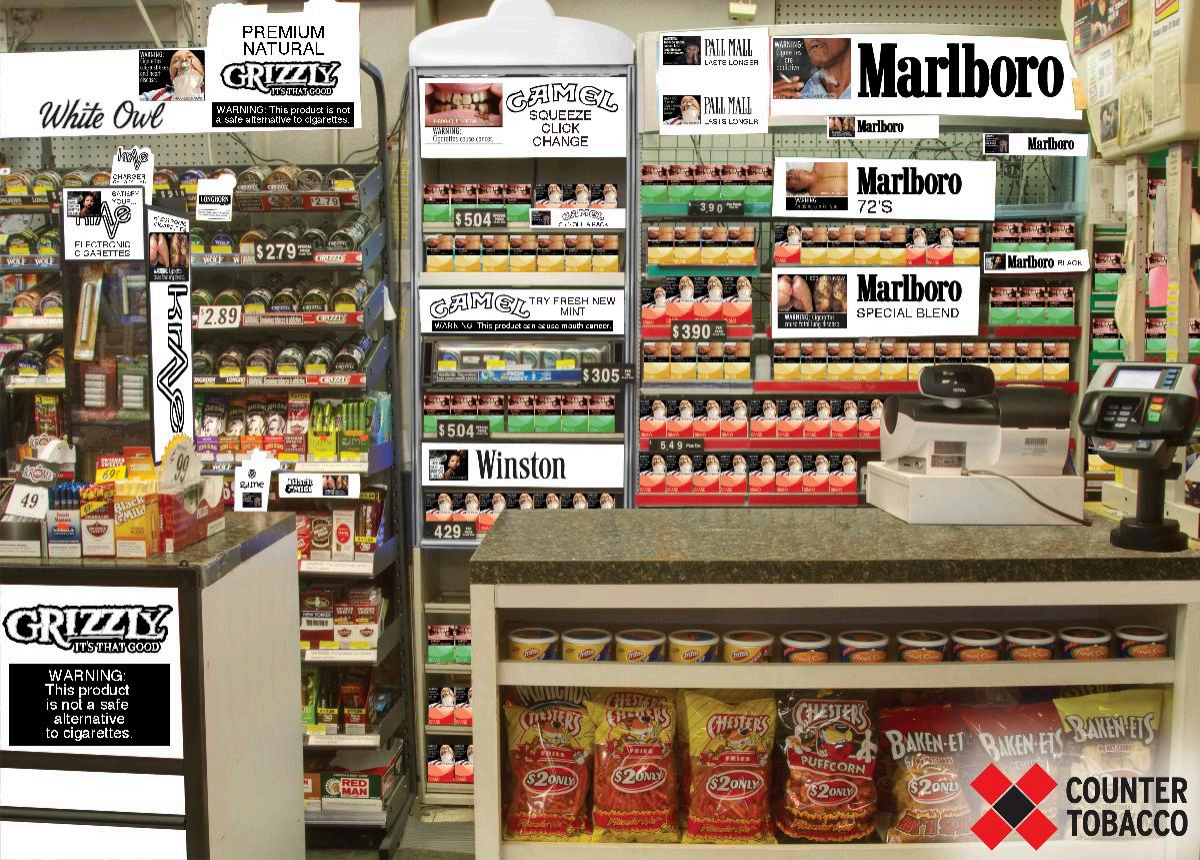
Illustration demonstrating a change to black and white (“tombstone”) tobacco advertisements with potential FDA warning messages covering 20% of advertisements and displayed on cigarette packs (after).

The Tobacco Control Act would mandate larger and stronger graphic warning labels, which may feature diseased lungs and other body parts, to appear on both cigarette packs and advertising (Figure 1). The graphic warning labels on packs and advertisements grab the viewer’s attention and will feature images many smokers and customers will find disturbing. Although the size of graphic images on *packs* may appear small to consumers, most tobacco *advertisements* are fairly large and easily visible. An overhead merchandising unit at the cash register or a large sign that might measure 10 square feet would require a graphic warning covering 20% of the advertisement. Imagine the visual impact if graphic warnings were expanded up to 2 square feet (Figure 2).

Despite setbacks due to industry litigation (Discount Tobacco and Lottery v. United States, Nos. 10–5234 and 10–5235, 2012 W.L. 899073 [6th Cir. March 19, 2012]), the Tobacco Control Act graphic warning labels, combined with stronger state and local regulations resulting from the lifting of federal preemption over regulations governing the time, place, and manner of tobacco product advertising (12), will likely result in fewer tobacco advertisements displayed in retail outlets. In addition, retailers may free up shelf space previously allocated to cigarettes, given declining smoking rates and lower consumer demand.

### Opportunities for combining tobacco control and obesity prevention

Tobacco retailers face the reality of a declining cigarette market (13). CVS Caremark stopped selling tobacco products because, as a retail pharmacy and clinic, the “financial gain is outweighed by the paradox inherent in promoting health while contributing to tobacco-related deaths” (14). Tobacco products were sold at CVS in a prime location behind the checkout counter and their removal creates an opportunity for other, healthier products and messages to fill the void. Although beverages, candy, and snack foods typically yield high profit margins (15), an opportunity lies in the growing “better-for-you” food and beverage product category, which includes low calorie and healthier options like whole grain cereal and yogurt (16). It is essential to ensure that junk food (ie, foods high in calories and that usually have added sugar and fats, such as candy, cookies, and potato chips) and sugar-sweetened beverages do not replace tobacco behind the counter and that systematic efforts are made to promote the consumption of healthful foods and beverages, such as fruits, vegetables, and water.

Public health practitioners working with retailers can capitalize on policy changes to the tobacco retail environment coupled with industry and consumer trends showing the public’s greater interest in healthful products. We summarize regulatory changes either mandated by the Tobacco Control Act or permitted by states, given the lifting of federal preemption over regulations governing the time, place, and manner of tobacco product advertising (12) (Table 2). Table 2 also shows how to capitalize on these changes to promote healthful eating and active living through improved neighborhood food and physical activity environments.

**Table 2:** Actual and Potential Local, State, and Federal Regulatory Changes Affecting Tobacco Retailers and Implications for Efforts to Promote Healthy Eating and Active Living

Regulatory Change	Implication
Requirement of graphic pictorial warning appearing on cigarette packages.	Stores may attempt to “hide” cigarette packs and place them out of sight, creating new merchandising space for healthy food behind the primary selling counter.
Ban or partial restriction on the visible display of tobacco products.	Tobacco products will be behind opaque shelving, presenting new opportunities for signage to promote healthy foods on the outside of shelving.
Requirement of graphic pictorial warning appearing on cigarette advertisements.	Stores may remove cigarette advertisements, providing new space for advertising healthy food options and healthy foods.
Removal of self-service display racks for cigars, e-cigarettes, and other tobacco products.	Racks can be used for selling healthy food options.
Banning of tobacco product sales at pharmacies.	More display space for tobacco use cessation products (eg, nicotine replacement therapy) and healthy foods.
Retailer reduction: reducing the number, type, and density of tobacco product retailers.	Retailers stop carrying tobacco products as a product line and become small convenience stores stocked with healthy food options.
Banning or restricting outdoor cigarette and smokeless tobacco product advertising near schools and parks.	Conversion of existing outdoor signage to promote healthy food options and physical activities at schools and parks.

### Implications for research, policy, and practice

Two major areas of future research and practice could inform the transition process for tobacco retailers. First, store observation studies could simultaneously collect information on the consumer tobacco, food, and physical activity environment adjacent to the store. This research would allow for an integrated assessment of the role of the consumer environment in contributing to 3 major risk factors for cancer and cardiovascular disease: tobacco use, poor dietary behaviors, and physical inactivity. For example, the food, tobacco and physical activity environments within and around retail stores were jointly examined in North Carolina (17), and an effort in California integrated tobacco, food, and alcohol in-store audits (18). Aside from these efforts, multiple aspects of the consumer environment that influence health have not been studied jointly, and audit tools to conduct a joint assessment are lacking.

Similarly, communities could conduct joint assessments of their food, tobacco, and activity environments. Food deserts and food swamps (areas where energy-dense foods inundate healthful options) are primarily in racial/ethnic minority and low-income neighborhoods (19). Are these the same areas where tobacco retailers are highly clustered (“tobacco swamps”) and where there are few nearby options for physical activity? Empirical studies of the relationship between tobacco, food, and physical activity environments could contribute to our knowledge of how disparities in community and consumer environments might contribute to health disparities, and such studies could help communities map health impact zones for priority interventions or to inform local policies, zoning regulations, and licensing ordinances. This process could also detect areas where an opportunity exists to develop partnerships between public health groups and retailers. For instance, retailers may work with local farms, cooperatives, and community-supported agriculture programs to facilitate the transition toward selling healthier foods, including fresh produce.

Second, programs, policies, and interventions can capitalize on changes in the retail tobacco advertising environment to promote healthful food purchasing and physical activity. Current definitions of “healthy stores” do not always include restrictions on tobacco (or alcohol) advertising and no studies, to our knowledge, have examined the possibility of repositioning tobacco outlets as a venue not only for promoting healthful food but also for facilitating physical activity. Sallis and Glanz (20) identified a need for multilevel intervention studies that simultaneously address physical activity and food environments as risk factors for obesity. Tobacco farmers transitioned successfully from growing tobacco to growing other crops (21); tobacco retailers are contemporary candidates for similar transitions.

Improving access to high-quality, affordable fruits and vegetables in retail stores in underserved communities is 1 of 10 Centers for Disease Control and Prevention (CDC) strategies to increase fruit and vegetable consumption (22). Using the comprehensive healthy stores approach proposed in this article, in-store promotion and marketing of fruits and vegetables could be increased by using the space previously reserved for tobacco advertisements and marketing. This type of shift would translate into a double transition — moving away from prioritizing shelf and marketing space for unhealthful foods to promoting healthful foods, and moving from high dependence to low dependence on tobacco products.

Before committing to stocking and promoting new items, establishing customer demand and profitability for healthful foods and beverages is essential (4). For this reason, community engagement is critical, and healthy store programs or policy implementation may be more successful when devoted community members assist with the transition. Gaining community support will demonstrate to retailers that customers want and will purchase healthier items, which makes the transition less financially risky. The Healthy Corner Store Network (http://www.healthycornerstores.org) is an excellent resource for practitioners and advocates seeking assistance on implementing a healthy corner store initiative.

Evidence from both cross-sectional and intervention studies suggests that once stores stock more healthful items, customers would purchase them (4). The Philadelphia Healthy Corner Store Initiative enrolled 630 corner stores over 2 years and found that even stores receiving a low-intensity (“basic”) intervention still showed an increase in healthful food availability over time (23). In San Francisco, a healthful retail program found that even nonparticipating stores made changes to improve their healthful options and thereby improve their rating in a local shopping guide (18).

Successful store transitions will rely on community engagement and retailer buy-in, which could be achieved through incentive programs. CDC recommends financial (eg, tax incentives, grants to cover equipment costs) or nonfinancial (eg, technical assistance) incentives to retailers to provide healthier food and beverage options in underserved communities as a strategy to reduce obesity (22). Practitioners could broaden the definition of “healthy stores” beyond healthful foods to include consideration of both reducing tobacco products and promoting physical activity. State or local programs or licensing ordinances to improve healthful food availability within retail food outlets could be expanded to explicitly target tobacco retailers. Some model healthy retailer ordinances include requirements for compliance with tobacco-related laws and also provide incentives to retailers who voluntarily reduce tobacco products in their stores or eliminate them altogether (24). For example, stores receiving a “healthy store” tax incentive might be required to have no point-of-sale tobacco advertisements or perhaps 5 or fewer. Regulatory changes combined with local licensing ordinances that decrease space for tobacco product marketing could result in increased space for marketing healthful foods and beverages at the point of sale.

Another strategy is to encourage retailers to replace tobacco displays near the register with displays of healthful items. Healthy stores programs often provide shelving units or baskets as an incentive to stock and display healthful food items near the cash register (4). Displacing tobacco products with healthful food displays is the next step toward a healthier store and would reduce young people’s exposure to tobacco advertisements at the point of sale. Many tobacco retailers are located near schools (11), and nearly half of US adolescents reported visiting convenience stores at least once per week (25). In Baltimore, young people reported shopping frequently at corner stores near their homes and schools for snacks and candy (26). Retailers could also eliminate exterior signage for tobacco products and instead promote healthful foods and nearby recreational opportunities.

Adding an active living component to a healthy stores program could also help create safer routes to schools (saferoutespartnership.org). Many programs emphasize the importance of a clean, well-lit store exterior in attracting customers and even encourage retailers to install bike racks (24). Inside, the store offers the option to promote nearby physical activity resources and walking and bicycling routes leading to the store. Consideration must be given to incentives for the store owner to promote active living or to partner with local public health advocates and city planning staff to facilitate this process.

## Conclusion

Tobacco retail outlets have the potential to become a major venue for community intervention. The “Healthy Stores” movement is already actively working with convenience stores, most of which sell tobacco products. Much of the work at the point of sale is being conducted in isolation by advocates for tobacco control, improved nutrition, and increased physical activity. Interdisciplinary efforts are needed to simultaneously reduce the dependence of tobacco retailers on a deadly and declining product category and to increase their focus on selling and promoting healthful foods and creating environments conducive to active living. The goal of CDC’s Community Transformation Grants and Communities Putting Prevention to Work (CPPW) initiatives was to fund communities to address both obesity and tobacco use through multilevel, community-based interventions. Through the CPPW initiative, 85% of communities chose to implement a “point-of-purchase” strategy to increase the promotion and display healthful foods, 64% chose to promote physical activity opportunities through increased signage, and 77% of communities focused on a point-of-purchase tobacco strategy, such as restricting tobacco product advertising (27). Thus, the proposed comprehensive approach to creating healthful stores is responsive to the broad CDC goals and is in line with a movement toward healthier communities in the United States and rest of the world.

A final benefit of intervening with retailers is to address health disparities, given that more tobacco retailers, fewer healthful food options, and fewer physical activity resources are co-located in low-income and racial/ethnic minority neighborhoods (9,19,20). Practitioners will need to act quickly to capitalize on point-of-sale tobacco control policy changes. When tobacco product advertisements and displays are removed, a host of unhealthful products, including soft drinks, alcohol, and energy-dense snack foods, will be eager to fill the void.
